# Reinforcement and Curriculum Learning for Off-Road Navigation of an UGV with a 3D LiDAR

**DOI:** 10.3390/s23063239

**Published:** 2023-03-18

**Authors:** Manuel Sánchez, Jesús Morales, Jorge L. Martínez

**Affiliations:** Institute for Mechatronics Engineering and Cyber-Physical Systems, Universidad de Málaga, 29071 Málaga, Spain

**Keywords:** reinforcement learning, off-road navigation, curriculum learning, 3D LiDAR, unmanned ground vehicles, traversability, robotic simulations

## Abstract

This paper presents the use of deep Reinforcement Learning (RL) for autonomous navigation of an Unmanned Ground Vehicle (UGV) with an onboard three-dimensional (3D) Light Detection and Ranging (LiDAR) sensor in off-road environments. For training, both the robotic simulator Gazebo and the Curriculum Learning paradigm are applied. Furthermore, an Actor–Critic Neural Network (NN) scheme is chosen with a suitable state and a custom reward function. To employ the 3D LiDAR data as part of the input state of the NNs, a virtual two-dimensional (2D) traversability scanner is developed. The resulting Actor NN has been successfully tested in both real and simulated experiments and favorably compared with a previous reactive navigation approach on the same UGV.

## 1. Introduction

Off-road navigation of Unmanned Ground Vehicles (UGVs) is a challenging task that requires the ability to avoid obstacles below and above the surface level, including overhangs [1]. This can be accomplished with classical approaches, such as potential fields [2], or with modern machine learning (ML) [3]. Nevertheless, these techniques present some issues: for the first approach, manual parameter tuning is required, whereas for the second, adequate labeled data has to be available for training.

Reinforcement Learning (RL) is an ML approach that focuses on directly mapping situations to actions to maximize a numerical reward [4]. In general, the training data for RL is generated when an agent interacts by trial and error with the environment through an action, that will produce a new state and a reward related to the achievement of a goal. RL has been applied to a wide range of areas, such as games [4], computer vision [5] or robotics [6]. In the latter, RL has been successfully employed for manipulator control [7], for indoor navigation [8,9] and for self-driving cars [10,11], but little research has been made on off-road scenarios.

To apply RL to autonomous navigation, it is necessary to employ vehicle simulators [12], as training requires many hours and exposure to dangerous situations such as collisions or overturns. Furthermore, autonomous navigation in off-road environments implies abilities that are difficult to grasp and should be taught in an incremental way as in Curriculum Learning (CL) [13].

In this paper, we propose a deep RL scheme, that makes use of the robotic simulator Gazebo [14] and the CL paradigm, trains a pair of Neural Networks (NN), namely Actor–Critic [4,15], to perform autonomous navigation of a UGV on off-road environments. To this end, the input data is the two-Dimensional (2D) traversability data obtained from tri-Dimensional (3D) point clouds of an onboard Light Detection and Ranging (LiDAR) sensor, and the relative position and orientation of the UGV with respect to the current geodetic goal. This navigation strategy has been tested in simulated and real scenarios, including a comparison with a previous reactive approach on the same UGV.

In this way, the main contributions of the paper can be summarized as follows:

- Generation of virtual 2D traversability ranges from a 3D laser scan, using a Random Forest (RF) classifier trained with synthetic data.
- Implementation of an Actor–Critic RL scheme, which has been trained in simulated scenarios of increasing difficulty with CL.
- Testing autonomous navigation on natural environments with the Actor NN in both simulated and real experiments.

The rest of the paper is organized as follows. The next section presents some related work. Then, Section 3 introduces the development of virtual 2D traversability scans from the 3D LiDAR data of the mobile robot Andabata. Section 4 describes the characteristics of the employed RL scheme. The training process and the experimental tests are shown in Section 5 and Section 6, respectively. The paper ends with a section devoted to conclusions and the cited references.

## 2. Related Work

### 2.1. Non-Trained Methods

For end-to-end navigation, it is generally required a method to estimate the traversability of the surroundings using onboard sensors of the UGV, such as LiDARs and cameras [16]. This task can be done by extracting different geometric features of the environment and using statistical analysis to estimate traversability. Thus, in [17] a 3D point cloud is processed to estimate feasible vehicle poses in the surroundings. In [18], a rapidly exploring random trees algorithm is directly used with 3D point clouds as input to produce safe paths within the mapped environment. In [19], fuzzy elevation maps are built from 3D LiDAR data to choose the best motion direction towards a distant goal. These methods require heuristic tuning that involves expert knowledge and is hard to transfer to other UGVs.

### 2.2. Data Trained Methods

Learning-based techniques have been successfully implemented for end-to-end navigation [20]. In [21], the robot behavior is learned using examples of the desired navigation in complex unstructured scenarios. As these kinds of tests with real robots are time-consuming and can affect robot integrity, the use of robotic simulations is very extended, like in [22], where traversability data is obtained with Gazebo and, then, used for navigation. In [23], an ML method is trained to classify point clouds of rough terrain using geometric information. In [24], different ML estimators are trained with synthetic LiDAR data, and the best classifier is applied in [25] to 3D point clouds to calculate a safe path towards the goals. Nevertheless, finding suitable data to train these algorithms is not always easy, as they usually require manual tagging, which is a slow and error-prone process. To overcome this problem, synthetic datasets have been published, where data is directly acquired from a robotic simulator [26], making it free of labeling errors and reducing human labor.

#### 2.2.1. Reinforcement Learning (RL)

One step further in data-trained algorithms for autonomous navigation is the use of RL. In this case, the training data is generated while practicing, and the behavior of the vehicle is continually adjusted [4]. Thus, in [27], obstacle avoidance is learned by a UGV while performing 2D path tracking. In [28], an unmanned aerial vehicle is trained with Gazebo to fly among obstacles with a 2D LiDAR. For indoor navigation of UGVs, methods can be found where the main exteroceptive sensors are a 2D rangefinders [29,30,31], depth cameras with a limited field of view [32] or RGB cameras [9,33]. In [34], 2D virtual range data generated from a monocular camera is employed by a UGV as input for RL. In [35], an onboard 3D LiDAR is used to build local elevation maps for navigating rough terrain with RL.

#### 2.2.2. Curriculum Learning (CL)

When facing complicated tasks, it is often beneficial to employ the CL paradigm, where the training process is done in different stages of increasing difficulty [13]. Thus, in [36] a robotic arm learns to grasp and place objects. In [37], a UGV finds out how to perform end-to-end navigation in warehouses with 2D LiDARs and a frontal camera. In [38], autonomous car driving is trained with the CARLA simulator [10] from a static environment with no traffic to more realistic settings with cars, pedestrians and changing weather conditions.

Finally, it is relevant to mention that most of the previously cited papers about RL adopt an Actor–Critic scheme [9,27,28,29,31,33,35,36,37,38].

## 3. Virtual 2D Traversability Scanner

Modern 3D LiDAR sensors produce a variable and large amount of 3D points, making this kind of data unsuitable for being used as input for RL, which requires a small-size constant-length vector. For this reason, 3D point clouds will be processed to emulate the output of a virtual 2D traversability scanner.

### 3.1. Case Study

**Figure 1:**
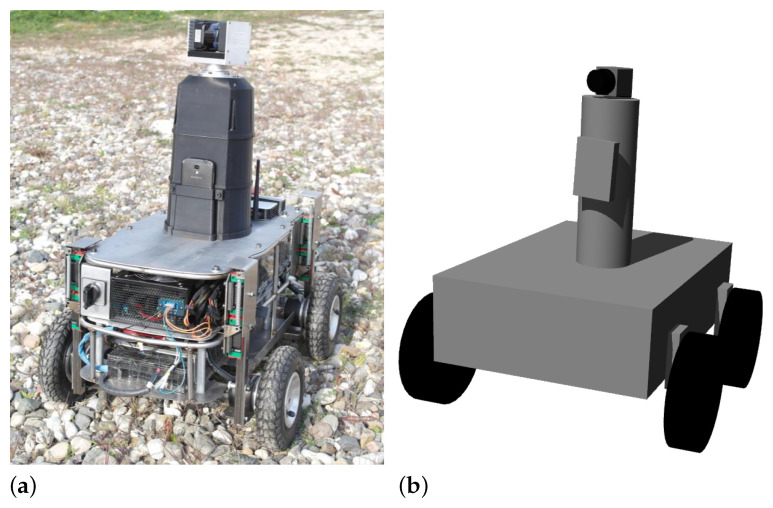
The mobile robot Andabata (**a**) and its simplified hardware model in Gazebo (**b**).

Andabata is a wheeled skid-steer UGV designed for off-road navigation that weighs 41 kg. It is 0.67 m long, 0.54 m wide and 0.81 m height (see Figure 1a). It has four DC motors (one for each wheel) powered by a 30 V battery [19]. The local coordinate frame is placed at the center of the wheel contact points with the ground with its local X, Y and Z axes pointing forward, to the left and upwards, respectively.

For outdoor localization, Andabata employs the IMU of a smartphone (with inclinometers, gyroscopes, and a compass) and a GNSS receiver with a horizontal resolution of 1 m. The main exteroceptive sensor for navigation is a 3D LiDAR sensor with 360° field of view built by rotating a 2D LiDAR [39].

All motors and sensors are connected to the onboard computer (Intel Core processor i7 4771 with 16 GB RAM, 8 MB cache and four cores at 3.5 GHz), which employs Ubuntu 18.04 and the Melodic version of the Robot Operating System (ROS). The hardware and software of Andabata have been modeled in Gazebo (see Figure 1b) to perform reliable simulations [25].

### 3.2. Traversability Assesment

In [24], a method to directly process the 3D leveled point clouds generated by Andabata was presented, where every 3D point was classified according to its traversability. Different classifiers were trained using supervised learning with labeled data generated synthetically. Then, binary classified points were projected in a local 2D horizontal grid to determine cell traversability for autonomous navigation [25].

In this paper, a simplified version of these procedures is proposed. Firstly, the 3D points are projected on a horizontal plane at the current position of the vehicle, and a local 2D polar grid is built, which consists of 32 sectors of 11.25° and 10 uneven annuluses with a maximum radius of 10 m as in [25]. 3D points with a height greater than 1.2 m are not projected, which would allow Andabata to move underneath overhangs such as tree canopy.

Then, five geometric features are calculated for every cell with the spatial coordinates of the 3D points that fall inside it: roughness ($F_{1}$), vertical orientation ($F_{2}$), planarity ($F_{3}$), the minimum height ($F_{4}$) and the maximum difference in height ($F_{5}$). The first three features are calculated by Principal Component Analysis (PCA).

**Figure 2:**
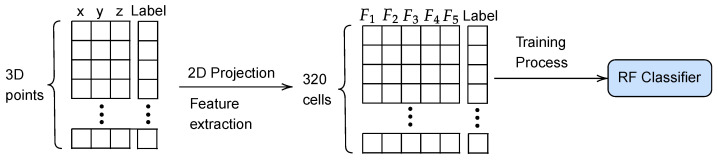
Pipeline for training the RF cell classifier.

**Figure 3:**
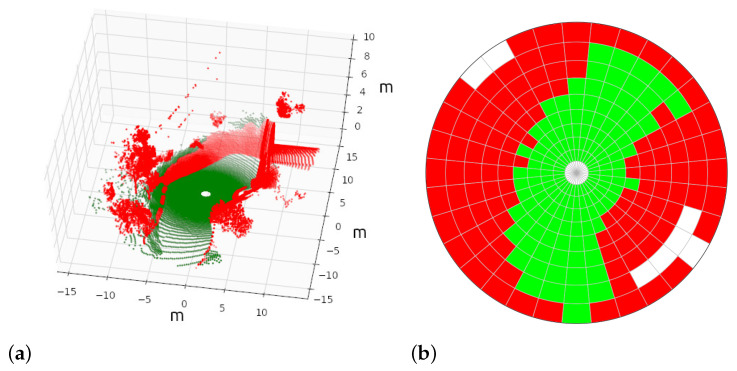
A synthetic 3D point cloud (**a**) and its tagged 2D polar grid (**b**) for training the RF cell classifier.

A new RF classifier is trained to assess the traversability of each cell (see Figure 2). This is done by using synthetic 3D scans with every point already labeled as traversable and non-traversable (see Figure 3a). Then, each polar cell is tagged according to the following criteria (see Figure 3b):

- If the cell does not contain enough points to compute geometric features (i.e., five), it is labeled as empty in white.
- If at least 15% of points are non-traversable, the cell is classified as non-traversable in red.
- In another case, i.e., with more than 85% of traversable points, the cell is classified as traversable in green.

The same fifteen synthetic 3D point clouds used in [24] have been employed for supervised learning, where ten of them are for training and five only for validation. All in all, this data contains 4800 cells, from which 2922, 1191 and 687 are green, red and white, respectively. Table 1 contains the components of the confusion matrix for the validation data by considering the negative and positive green and red classes, respectively. Performance metrics have been computed in Table 2, where good classification results can be observed, although slightly worse than those obtained for the RF point estimator [24].

**Figure 4:**
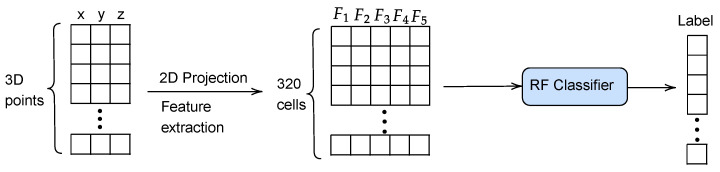
Pipeline for the prediction of cell traversability.

Once trained the RF classifier, can be employed for predicting the traversability label of the 2D cells of a 3D point cloud (see Figure 4). This constitutes an improvement in the processing time with respect to our previous navigation strategy [25], because it directly classifies 320 navigation cells instead of around 32,000 3D points.

**Figure 5:**
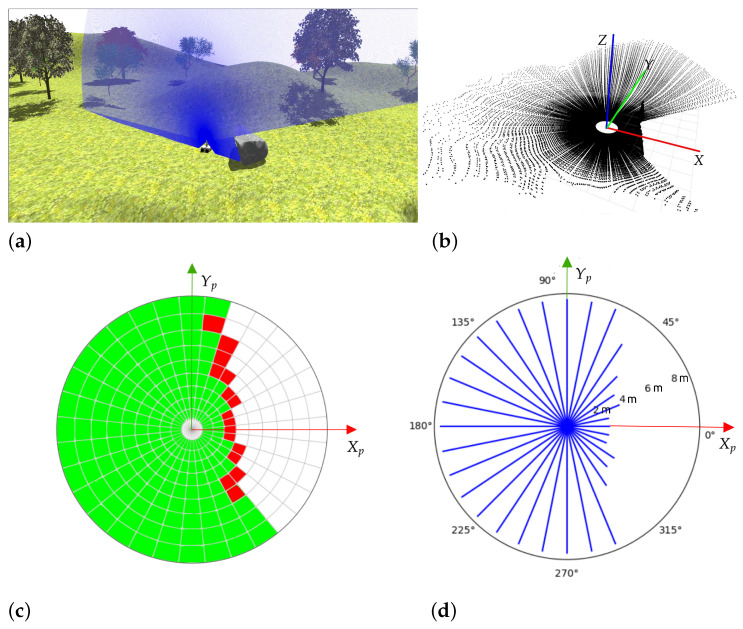
Visualization of a single vertical 2D scan from the simulated 3D LiDAR of Andabata (**a**), a 3D point cloud (**b**), the deduced cell traversability (**c**) and the virtual 2D traversability ranges (**d**).

Finally, with the traversability grid, the virtual 2D scanner will produce a vector $v_{t}$ at the discrete time step *t* with 32 maximum navigable distances along every sector. An example of this processing on the simulated environment is shown in Figure 5, where $X_{p}$ and $Y_{p}$ represent the projection of the local *X* and *Y* axes of Andabata on the 2D horizontal grid, respectively.

## 4. Deep Reinforcement Learning

**Figure 6:**
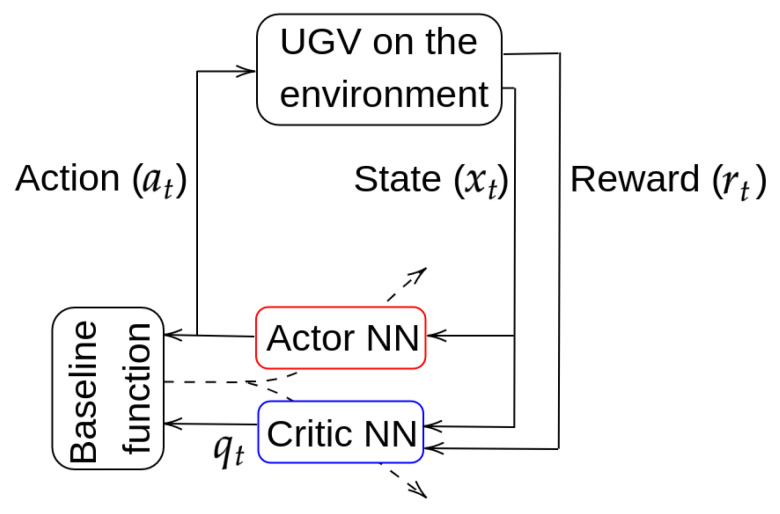
Actor–Critic general scheme.

For deep RL, it has been chosen a model-free, on-policy and online scheme, called Actor–Critic [4,15], that tries to adjust, at the same time, the Actor and the Critic NNs. During training, the Actor selects an action $a_{t}$ for a given state $x_{t}$, whereas the Critic informs how good was the selected action with a value $q_{t}$. A baseline function, calculated with $a_{t}$ and $q_{t}$, serves to adjust both NNs during training (see Figure 6).

**Figure 7:**
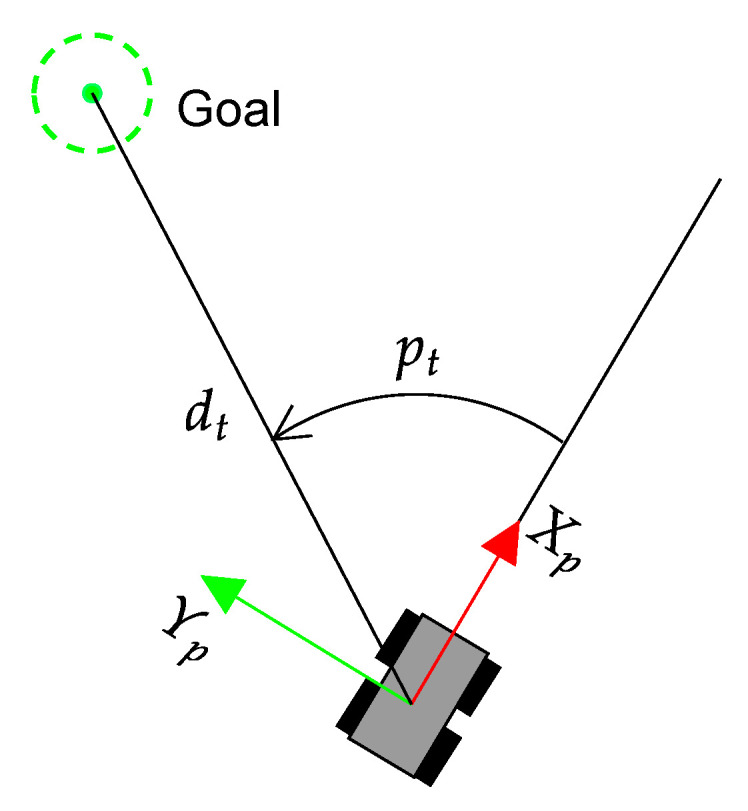
Heading error $p_{t}$ and distance $d_{t}$ of Andabata with respect to the target at the discrete time step *t*.

The state $x_{t}$ has 35 values that consist of the traversability vector $v_{t}$, together with two previous actions (indicated as $a_{t-1},a_{t-2}$) and the heading error $p_{t}$ of the UGV with respect to the current goal (see Figure 7), which is obtained from the GNSS coordinates and compass orientation. The action is directly the steering speed of Andabata, which is in the domain $a_{t}\in \left[-0.5,0.5\right]$ rad/s and includes a dead zone of [−0.15,0.15] rad/s to avoid small changes of direction.

**Figure 8:**
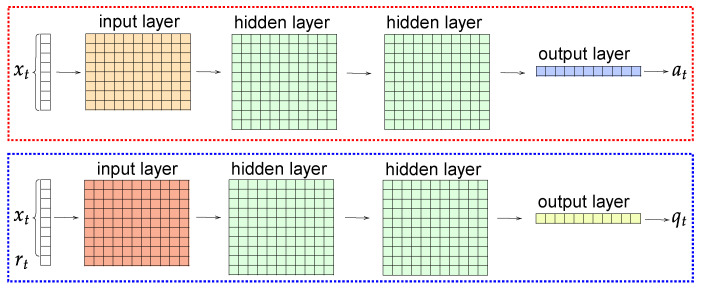
Layers of the Actor (**top**) and the Critic (**bottom**) NNs.

The Actor and Critic linear NNs each contain two hidden square layers (200 × 200 dimension) and an output layer (200 × 1 dimension) which have been implemented by using the ML library PyTorch (https://pytorch.org, accessed on 14 March 2023). The dimensions of the input layers for Actor and Critic NNs are 35 × 200 and 36 × 200, respectively. Both have as input $x_{t}$, but the Critic NN also includes the environmental reward $r_{t}$ (see Figure 8).

### 4.1. Reward Function

**Figure 9:**
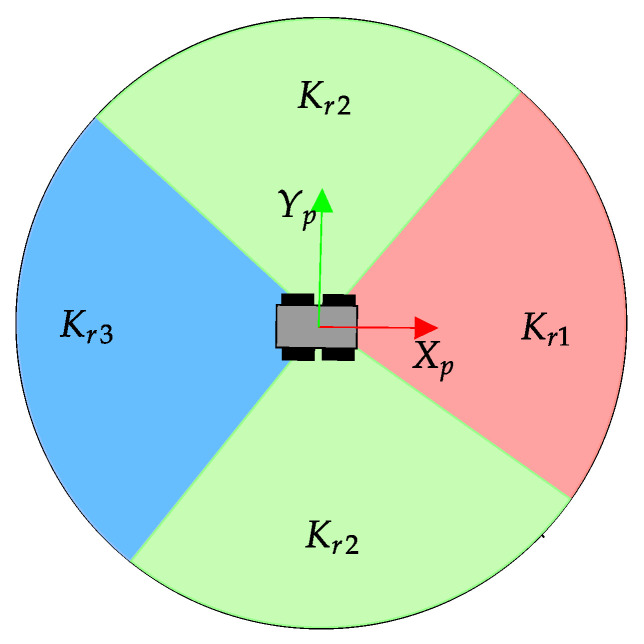
Possible values for the $K_{r}$ parameter.

The reward function $r_{t}$ evaluates the situation of the UGV in the environment. It has been adjusted by trial and error as:

(1) $$r_{t}=r_{t}^{a}-r_{t}^{r},$$

where $r_{t}^{a}$ and $r_{t}^{r}$ are the attractive and repulsive functions, respectively. The attractive function is defined as:

(2) $$r_{t}^{a}=K_{a}\;\cdot \;\left(d_{t}-d_{t-1}\right),$$

where $K_{a}=200$, $d_{t}$ and $d_{t-1}$ are the distances between Andabata and the goal point at the current time step and at the previous (see Figure 7). The repulsive function is modeled as:

(3) $$r_{t}^{r}=\sum_{i=1}^{32}K_{r}\left(i\right)\;\cdot \;\left(v_{max}-v_{t}\left(i\right)\right),$$

where *v_max_* = 10 m, $v_{t}\left(i\right)$ is the 2D virtual range in the direction *i* of the navigation grid and $K_{r}$ a parameter that depends on the sector that corresponds to *i* (see Figure 9). Three values $K_{r1}=0.1$, $K_{r2}=0.03$ and $K_{r3}=0.009$ are chosen to produce a higher punishment when Andabata directly navigates to obstacles.

This strategy produces smoother steering behaviors than sparse penalty functions [29,31], where only a minimum distance to obstacles is considered for negative rewards, which can lead to rough UGV reactions.

### 4.2. ROS Implementation

**Figure 10:**
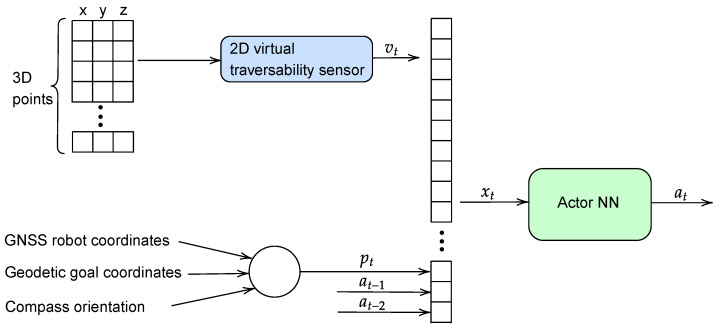
Inputs and outputs of the Actor NN during autonomous navigation.

Figure 10 shows the generation of a control action from a 3D point cloud through the trained Actor NN, that will allow Andabata to move towards a goal while avoiding obstacles in off-road environments.

**Figure 11:**
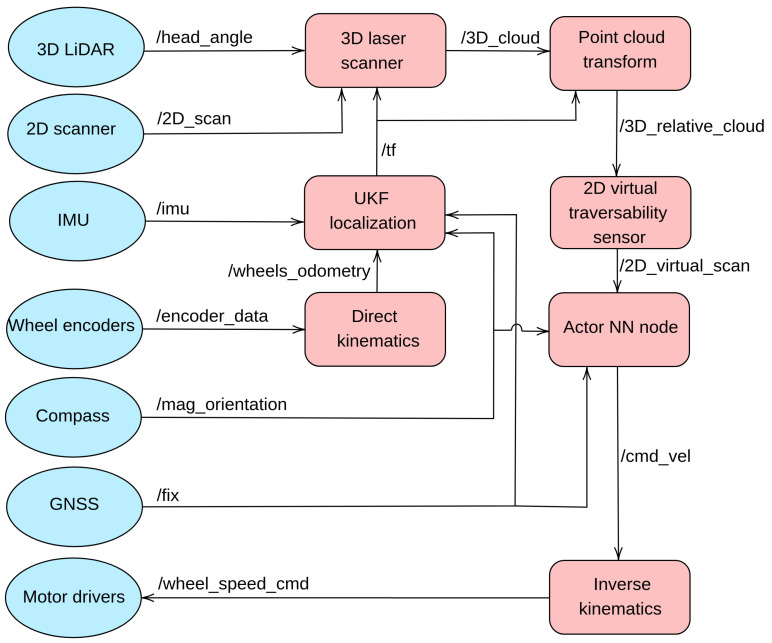
ROS computation graph. Driver nodes (Gazebo plugins for the simulated robot) are represented with blue ellipses instead of red squares.

The software necessary to perform autonomous navigation has been developed with ROS nodes, which are fully compatible between Andabata and its Gazebo implementation [25]. Figure 11 shows the ROS implementation in terms of nodes and topics. Table 3 shows the rates at which the different messages are delivered between different nodes in an asynchronous way through the topics. It stands out that the interval among control actions is 0.1 s, although the acquisition time for 3D laser scans is approximately 3.33 s.

The Unscented Kalman Filter (UKF) node generates the mobile robot localization by using the IMU, the compass and wheel odometry produced by the direct kinematics node. The 3D laser scanner node builds leveled 3D point clouds by combining vertical 2D laser scans with the angular position of the LiDAR head and UKF localization. To simulate the data generated by the real sensors and the motor drivers, several Gazebo plugins were introduced. All these nodes have been previously employed in [19].

While a new 3D point cloud is forming, the current one is continually transformed into the current robot pose. Then, these relative point clouds are processed to generate the 2D traversability data that the Actor NN node employs to produce angular speed commands. Finally, the inverse kinematics node computes the wheel speeds of each side for skid-steering assuming that the vehicle navigates at a constant longitudinal speed.

## 5. Training with Curriculum Learning

Following the CL strategy, three simulated scenarios of increasing complexity have been used to accelerate the training process and to increase its convergence. In this way, Andabata begins with a horizontal 2D laser scanner in an indoor scenario, continues with its 3D LiDAR and ends in a natural environment. In each stage, 200 navigation episodes are performed. Each one can be aborted either by reaching the goal (i.e., if $d_{t}$ < 1 m), when a collision is detected (i.e., if any of the elements of $v_{t}$ is less than 0.72 m) or when a maximum time is exceeded.

### 5.1. First Stage

**Figure 12:**
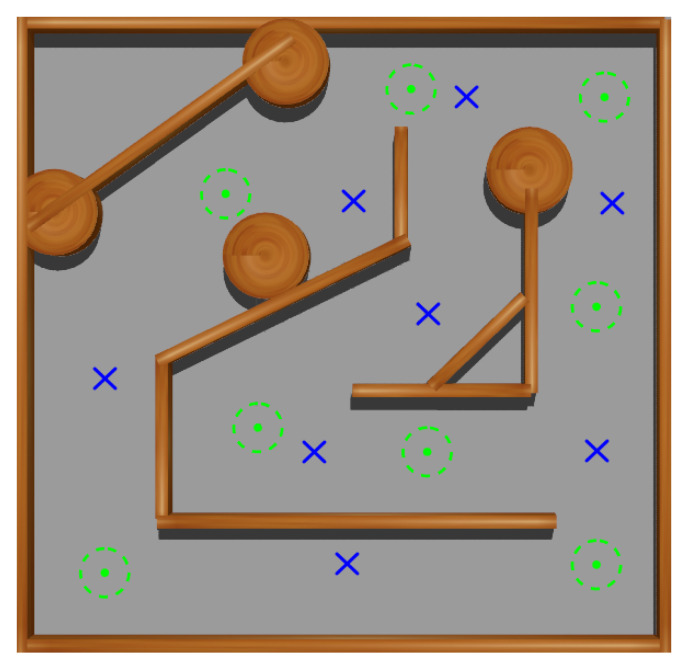
Top view of the training environment for the first and second stages. Possible initial and goal points are marked with blue crosses and green dots, respectively.

At this stage, a squared maze-like environment of 80 m side has been used. Several possible initial and goal positions for Andabata have been chosen for training the NNs (see Figure 12). The initial heading of the vehicle is always zero, i.e., it points to the upper side of Figure 12.

**Figure 13:**
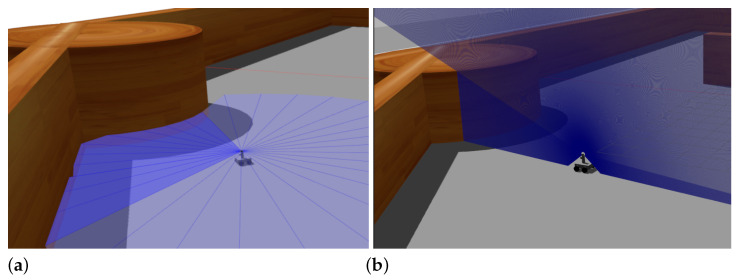
Examples of 2D laser scans acquired in the first (**a**) and in the second (**b**) stages in the maze.

In addition, the 3D LiDAR has been replaced by a 2D laser scanner with 32 horizontally mounted beams with a maximum range of 10 m. This is also the distance returned when obstacles are far away to emulate the 2D traversability virtual scanner (see Figure 13a). The maximum allowed time for each episode is 180 s.

**Figure 14:**
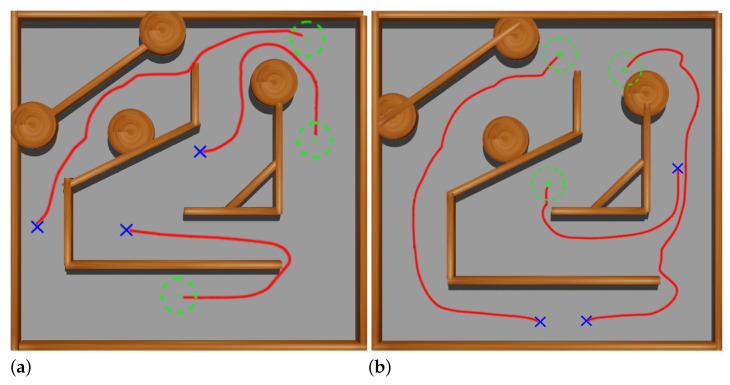
Validation tests at the end of the first (**a**) and second (**b**) stages. The followed paths are represented with red lines.

Figure 14a shows three tests using the trained Actor NN at this stage, where it can be observed good navigation results with a longitudinal speed of 0.3 m s^−1^ between initial and goal points.

### 5.2. Second Stage

At this stage, virtual 2D traversability scans are generated from the simulation of the 3D laser scanner of Andabata, which combines successive vertical 2D scans acquired in motion (see Figure 13b). The remaining navigation conditions from the previous stage have not been changed.

The main difference from the previous stage is that the ranges of the 2D virtual scanner are discontinuous rather than continuous. Three tests were done to examine the resulting Actor NN. Figure 14b shows the trajectories followed by Andabata, which correctly reach the goals while avoiding the walls of the maze.

### 5.3. Third Stage

**Figure 15:**
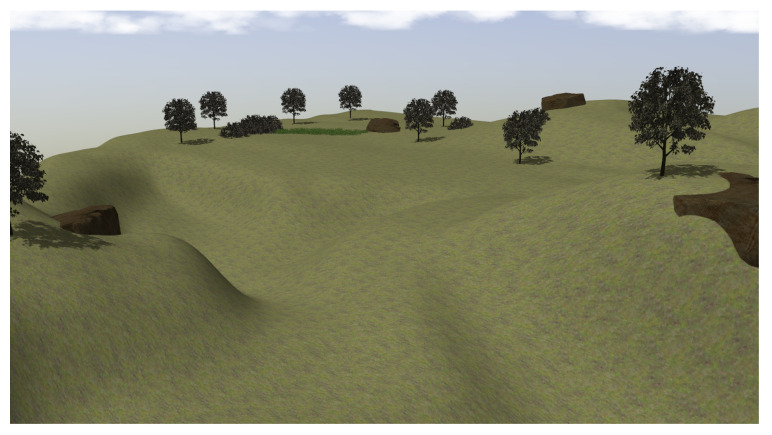
View of the modeled natural environment for training at the third stage.

At the last stage, the maze is substituted by a natural environment modeled with a Gazebo in a square of 120 m side that contains trees, high grass and bushes on uneven terrain with a maximum difference in height of 14 m (see Figure 15).

**Figure 16:**
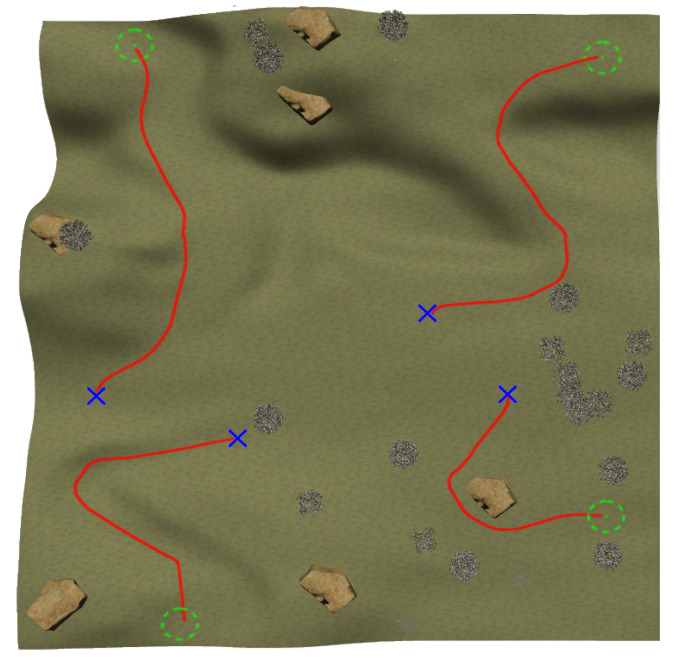
Navigation tests after the third training stage.

The NN training process continues with navigation episodes along four pairs of feasible initial and end positions with a maximum allowed time of 300 s. Figure 16 shows the paths followed among these pairs once finished training, where it can be observed that the mobile robot successfully reaches the goals while avoiding both negative and positive obstacles.

**Figure 17:**
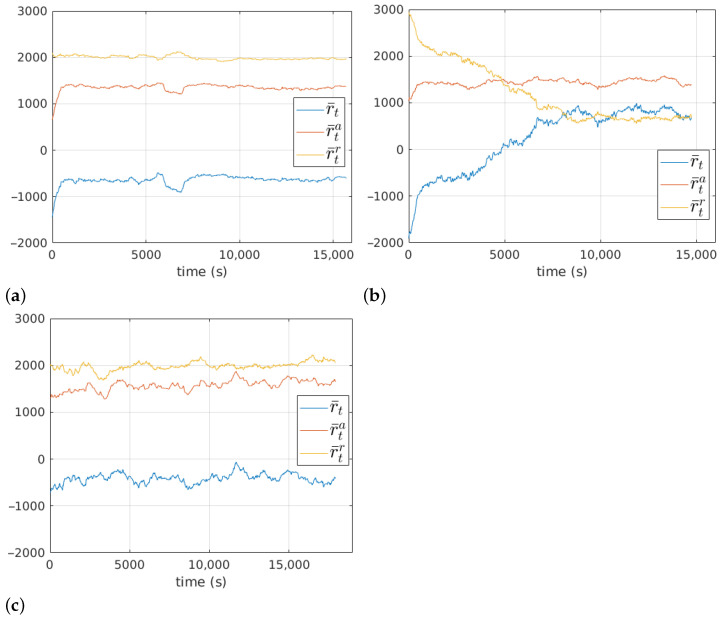
Evolution of the reward function at the first (**a**), the second (**b**) and the third (**c**) stages.

The moving average with 1000 time steps of the reward function along the 200 training episodes ${\bar{r}}_{t}$ is shown in Figure 17. The average duration of each episode was 83.4 s, 78.6 s and 94.6 s for the first, second and third stages, respectively. In the first stage, it can be observed that learning is achieved very quickly by improving ${\bar{r}}_{t}^{a}$. In the second stage, improvements require more training steps and involve the reduction of ${\bar{r}}_{t}^{r}$. The third stage shows a slow and slightly positive evolution of ${\bar{r}}_{t}$.

## 6. Experimental Results

### 6.1. Real Test

**Figure 18:**
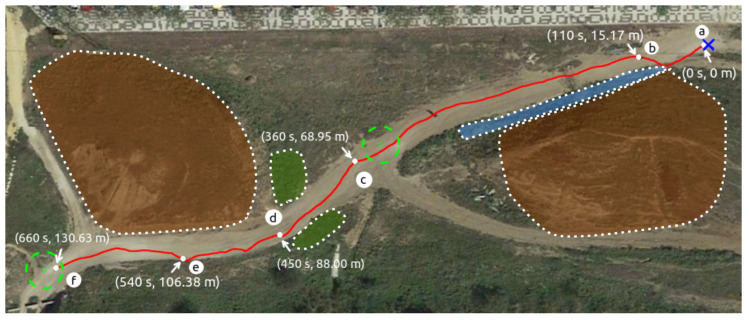
Path followed by Andabata with indications of traveled distance and elapsed time. Shadowed areas in brown, green and blue colors represent hills, weeds and ditches, respectively.

**Figure 19:**
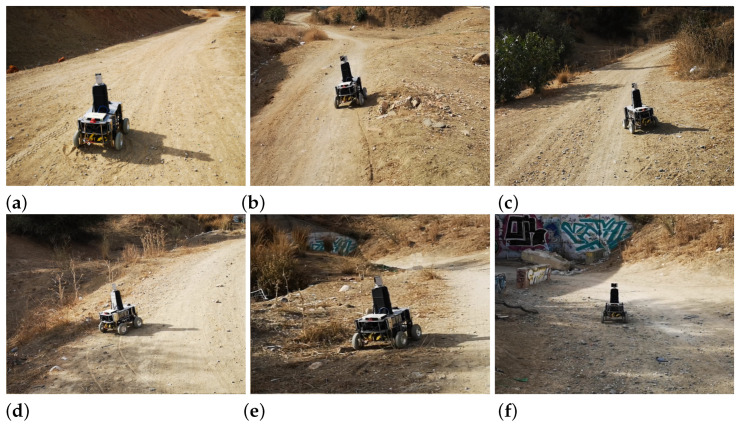
Photographs of Andabata during the autonomous navigation test at the spots indicated in Figure 18.

Using the trained Actor NN, a navigation test with Andabata was performed in a trail inside a hollow as in [25]. Figure 18 shows the path followed by the mobile robot, as recorded by the GNSS receiver, to reach two consecutive goals while avoiding weeds, hills and ditches. Figure 19 displays some snapshots along the experiment in the places marked in Figure 18 from the beginning (a) to the end (f) of the trajectory.

### 6.2. Simulated Test

**Figure 20:**
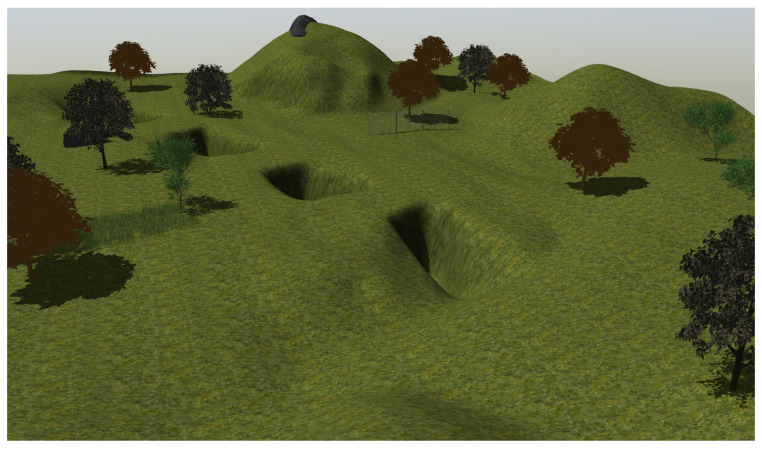
Modeled natural scenario for simulated testing [25].

An experiment has been performed on the natural environment already modeled in [25], which consists of a square of 100 m side with a maximum difference in height of 16 m (see Figure 20).

**Figure 21:**
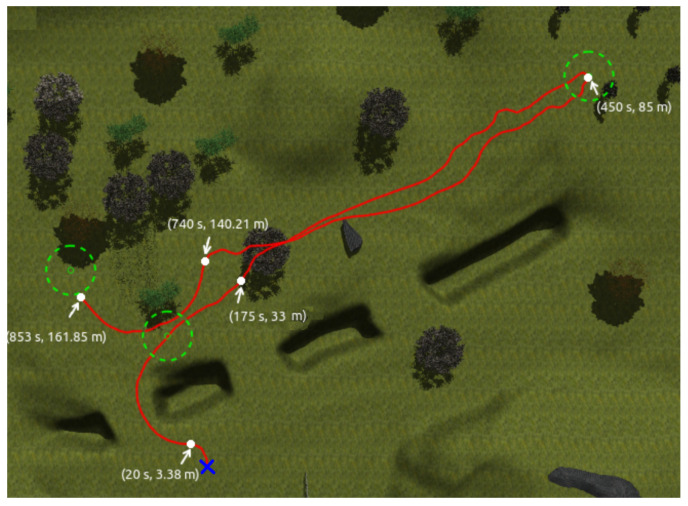
Path followed by the robot with time and distance stamps.

Figure 21 shows the path followed by Andabata while visiting three consecutive objectives. At 175 s, the mobile robot was able to avoid a negative obstacle, reach the first goal and point toward the second goal. Then, it reaches the second objective at 450 s and, on its way to the third goal, the vehicle avoids a high grass area among trees, starting this maneuver at 740 s.

### 6.3. Comparison between Reactive and RL Approaches

**Figure 22:**
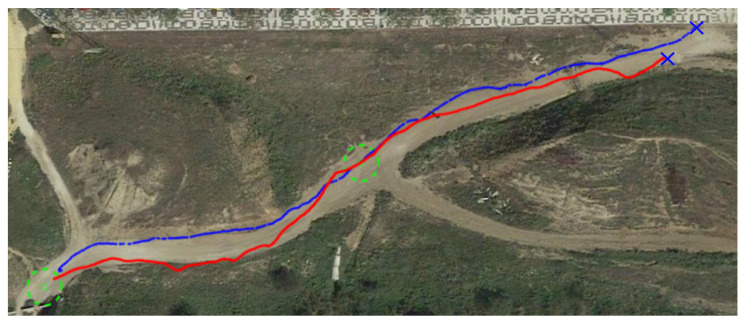
Comparison between reactive and RL paths on the hollow, represented with blue and red lines, respectively.

**Figure 23:**
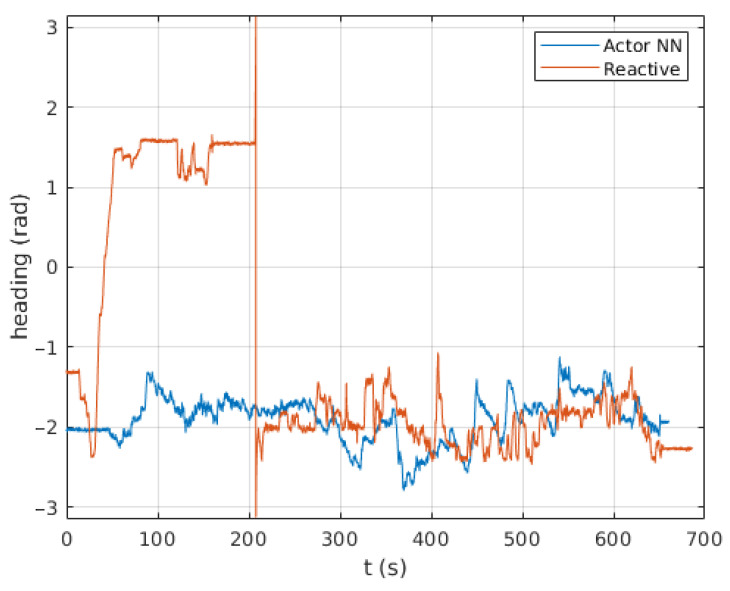
Heading changes along time with the reactive and the Actor NN controllers.

Following is a comparison between our previous reactive navigation method [25] and the proposed deep RL strategy. The paths tracked by Andabata on the real scenario are shown in Figure 22. The corresponding headings of the vehicle along these trajectories are represented in Figure 23. Although both methods have chosen different actions over time, no significant differences between both methods can be observed in these figures.

**Figure 24:**
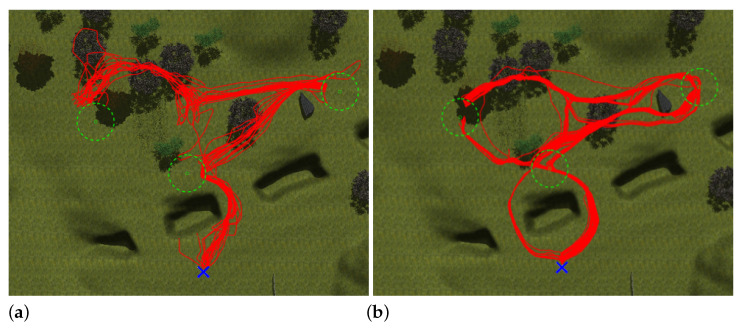
Paths followed by Andabata with the reactive (**a**) and the Actor NN (**b**) approaches.

To compare the success ratio between both approaches, a simulated test has been performed in the Gazebo environment of Figure 20. For this purpose, 50 navigation attempts have been made with the mobile robot to reach three consecutive objectives (see Figure 24). It can be observed that the Actor NN is capable of finding alternative paths that the reactive approach cannot. Table 4 shows the success percentage for each of these goals, where the Actor NN clearly displays better results.

## 7. Conclusions

The paper has presented the RL training process for off-road UGV navigation with an Actor–Critic scheme. This has been accomplished with CL by using realistic robotic simulations on scenarios of increasing difficulty. Virtual 2D traversability ranges, generated by an RF classifier from a leveled 3D laser scan, have been employed as input data for both NNs.

In this way, reactive navigation has been implemented for the Andabata mobile robot with its 3D laser scanner using ROS topics and nodes. One issue of the proposed deep RL method is that it requires time to retrain in case any parameter is changed, for example, on the reward function.

The resulting Actor NN has been successfully tested in both simulated and real experiments. In comparison with our previous navigation method on the same UGV [25], the resulting controller shows similar reactive behaviors, but with higher output rates of actions and increased reliability to reach successive goals.

Future work includes the employment of a commercial portable 3D LiDAR to improve the data acquisition rate. It is also of interest to generate more 2D traversability ranges and to consider the navigation time in the reward function, which is a limiting factor in battery-operated mobile robots.

## Figures and Tables

**Table 1:** Confusion matrix for the validation data.

Component	Value
True Positive (TP)	340
True Negative (TN)	780
False Positive (FP)	82
False Negative (FN)	210

**Table 2:** Validation metrics for the RF cell classifier.

Metric	Formula	Result
Precision	$\frac{TP+TN}{TP+TN+FP+FN}$	0.798
Recall (RE)	$\frac{TP}{TP+FN}$	0.618
Specificity (SP)	$\frac{TN}{TN+FP}$	0.906
Balanced Accuracy	$\frac{RE+SP}{2}$	0.762

**Table 3:** Output rate of the ROS messages.

ROS Topic	Message Type	Rate (Hz)
/2D_scan	sensor_msgs/LaserScan	40
/head_angle	andabata_msgs/LaserEvent	40
/encoder_data	andabata_msgs/Wheels_speed	100
/imu	sensor_msgs/Imu	100
/3D_cloud	sensor_msgs/PointCloud2	0.3
/3D_relative_cloud	sensor_msgs/PointCloud2	20
/tf	tf/tfMessage	1000
/mag_orientation	sensor_msgs/MagneticField	100
/fix	sensor_msgs/NavSatFix	1
/2D_virtual_scan	sensor_msgs/LaserScan	10
/cmd _vel	geometry_msgs/Twist	10
/wheel _speed _cmd	andabata_msgs/Wheels_cmd	10

**Table 4:** Success ratio for reaching three consecutive goals along 50 simulated trials.

Controller	Goal 1	Goal 2	Goal 3
Actor NN	98.0%	90.0%	70.0%
Reactive	80.0%	73.3%	53.3%

## Data Availability

Data sharing not applicable.

## Abbreviations

The following abbreviations are used in this manuscript:

2D	Two-Dimensional
3D	Three-Dimensional
CL	Curriculum Learning
FN	False Negative
FP	False Positive
GNSS	Global Navigation Satellite System
IMU	Inertial Measurement Unit
LiDAR	Light Detection Furthermore, Ranging
ML	Machine Learning
NN	Neural Network
PCA	Principal Components Analysis
RE	REcall
RL	Reinforcement Learning
RF	Random Forest
ROS	Robot Operating System
SP	SPecifity
TN	True Negative
TP	True Positive
UGV	Unmanned Ground Vehicle
UKF	Unscented Kalman Filter
